# Behaviour change and associated factors among Female Sex Workers in Kenya

**Published:** 2012-12-26

**Authors:** Josephat Nyagero, Samuel Wangila, Vincent Kutai, Susan Olango

**Affiliations:** 1 African Medical and Research Foundation (AMREF), Kenya

**Keywords:** Behaviour change, female sex workers, HIV prevention

## Abstract

**Background:**

Initiatives aimed at behaviour change of key populations such as the female sex workers (FSWs) are pivotal in reducing the transmission of HIV. An 8-year implementation research to establish the predictor factors of behaviour change among FSWs in Kenya was initiated by the African Medical Research Foundation (AMREF) with Sida and DfID support.

**Methods:**

This cross-sectional survey interviewed 159 female sex workers (FSWs) identified through snowball procedure. The measurement of behaviour change was based on: the consistent use of condoms with both regular and non regular clients, reduced number of clients, routine checks for STIs, and involvement in alternative income generating activities. The adjusted odds ratios at 95% confidence interval computed during binary logistic regression analysis were used to determine the behaviour change predictor factors.

**Results:**

Most FSWs (84%) had participated in AMREF's integrated intervention programme for at least one year and 59.1% had gone through behaviour change. The adjusted odds ratio showed that the FSWs with secondary education were 2.23 times likely to change behaviour, protestants were 4.61 times, those in sex work for >4 years were 2.36 times, FSWs with good HIV prevention knowledge were 4.37 times, and those engaged in alternative income generating activities were 2.30 times more likely to change their behaviour compared to respective counterparts.

**Conclusion:**

Behaviour change among FSWs was possible and is associated with the level of education, religious affiliation, number of years in sex work and one's level of HIV prevention knowledge. A re-orientation on the peer education programme to focus on HIV preventive measures beyond use of condoms is emphasized.

## Background

The role of female sex workers (FSWs) in facilitating transmission of sexually transmitted infections (STIs) including HIV is a subject of continued interest [1]. FSWs are considered a population group for the transmission of HIV and other sexually transmitted infections [1]. Regular partners or non-commercial partners of the female sex workers are another important risk group [1]. As such, FSWs are often categorised among the populations “most-at-risk” to HIV due to behaviours that heighten their exposure to the virus [2]. Due to the hazardous nature of their occupation, FSWs have a variety of concerns including; contracting sexually transmitted diseases (STDs) and HIV/AIDS, asthma, high blood pressure, and dying or getting killed on the streets [3]. Underlying risk factors for these include inconsistent condom use, presence of work-related violence, younger age and presence of non-paying sex clients [4]. The years of engaging in sex work have been associated with frequent acquisition of STIs, including HIV [4].

There is growing evidence that behaviour change interventions to reduce transmission levels of HIV among core groups can lead to successful risk reduction and decreased levels of infection. The interventions that have been used as a response to commercial sex work include; development and dissemination of behaviour change messages, promotion of condoms and other barrier methods, accessible sexual health services, use of informal contacts, key informants, and “leaders” to access the population, peer health promotion and education, outreach activities, condom social marketing and distribution, and income generating activities among others [5, 6]. Previous interventions targeting FSWs have yielded varied behaviour change across the world. In Ecuador, prostitution is legal and its practice requires that FSWs carry a stamped permit booklet and FSWs are required to present themselves for STI screening every 15 days [7]. This intervention has increased safety and decreased the risk of working in such establishments [8]. A study in Senegal [9] reported that 95% of the FSWs had undergone positive health behaviour change in comparison to the 40% level found in the Democratic Republic of Congo [10].

Previous studies have identified various determinants of behaviour change among FSWs. The knowledge and awareness of HIV and AIDS, perceived vulnerability, perceptions of outcomes including costs and benefits of condom use, social support, peer group comparison and condom use self-efficacy as key factors responsible for behaviour change among FSWs in India [11]. Population characteristics such as place of residence in the last two years, age, educational level and marital status have been reported as being important in determining the level of behaviour change [12]. The background characteristics (level of education, marital status and age) were similarly found to influence behaviour change among FSWs in north-western Tanzania [13] and Zambia [14]. Another study in Malawi found that the presence of sex worker peer educators led to increase in the use of condoms with paying clients [15]. Different environments are likely to influence individual country cohorts’ predictive factors; hence the need to study behaviour change among FSWs and its associated factors in Kenya.3

Since the late 1980s, African Medical and Research Foundation (AMREF) has been involved in implementation of interventions in Kenya, Tanzania and Uganda which aim to foster behaviour change among HIV high risk populations. This paper presents an assessment of behaviour change and its determinant factors among female sex workers (FSWs) participating in AMREF's Maanisha Programme. The programme works with civil society organisations (CSOs), private sector organisations (PSOs) and the Government of Kenya structures to achieve its goal of contributing to achievement of sustained reduction in the prevalence of HIV, AIDS and STIs in Kenya. It was launched in 2004. Maanisha programme targets people living with HIV and AIDS, female sex workers (FSWs), orphans and vulnerable children (OVC), men who have sex with men (MSM), youth, injecting drug users, widows and widowers and the general population. This paper focuses on project activities of FSWs.

## Methods

The conceptualisation of the study was guided by the Health Belief Model (HBM) and the Theory of Planned Behaviour which are widely used to predict behaviour [16]. The HBM proposes that people are only motivated to carry out preventive health behaviours as a response to a perceived threat to their health. Two classes of variables: first, the psychological state of readiness to take specific action, and second, the extent to which a particular course of action is beneficial in reducing the threat are the pillars of the model [16–18]. The Theory of Planned Behaviour presupposes that reasoned actions result from behavioural intentions that are largely based on one's attitudes and subjective norms [19].

**Figure 1:**
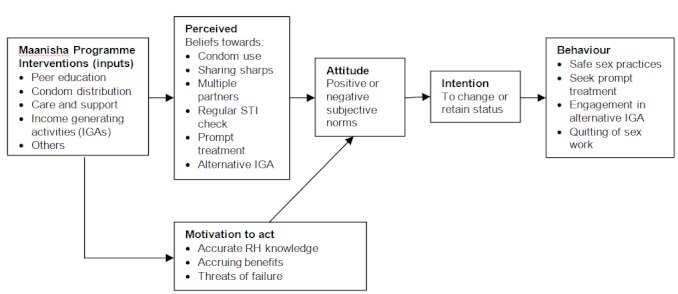
Theoretical framework of the study

The Maanisha Programme was designed to influence the behaviour of FSWs through a set of inputs including; peer education, condom distribution and income generating activities (IGAs), among others. The expected behaviour change is in terms of safer health practices (such as consistent use of condoms, reduced number of sex partners, non-sharing of sharps, regular seeking of STI medical checks and seeking of prompt STI treatment), and quitting the commercial sex work all together in favour of engagement in alternative sources of income. As proposed in the health belief model (HBM), the first group of variables focus on the psychological state of readiness to change behaviour considered in this study include; the FSWs’ beliefs towards condom use, sharing of sharps, having multiple sex partners, regular STI medical check, prompt STI treatment and engagement in alternative income generating activities (Figure 1).

In the second set of variables, this study presumes that behaviour change can be motivated through the provision of clear messages on the benefits accruing from the desired behaviour change being promoted and the threats of failure to adopt the change. The assumption here is that the resultant behaviour is based on positive attitudes and subjective norms whose formation may be significantly attributed to the set of AMREF project inputs and activities. Examples of expected benefits associated with motivation to act may include better health status and improved living standards for the FSWs. These factors assist in shaping attitudes and subjective norms resulting in shaping of intentions on decisions for behaviour change or retention of the status quo.

**Figure 2:**
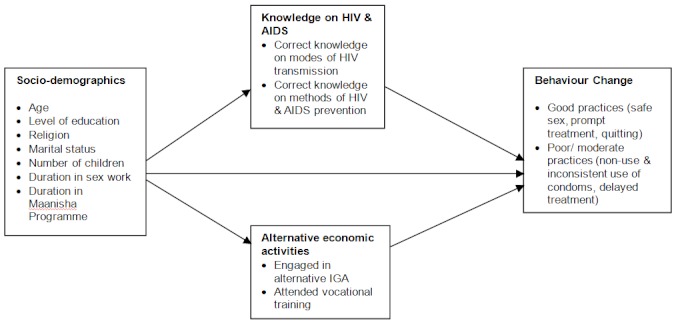
Conceptualisation framework of the study

This study tested whether behaviour change towards good health practices (dependent variable) among participating FSWs was influenced by their socio-demographic characteristics (age, level of education, religion, marital status, having children, duration in sex work and participation in AMREF project), the level of knowledge on reproductive health, HIV and AIDS, and participation in alternative economic activities. Figure 2 schematically illustrates this relationship.

Based on the relationship between variables, the study tested the null hypotheses that: socio-demographic characteristics, level of HIV and AIDS knowledge (partially gained through the project's peer education activities) and engagement of FSWs in alternative economic activities does not predict their behaviour change.

It was necessary to operationalise the definitions and measurement of key study variables. Behaviour change was defined as any new desired/ good behaviour associated with the prevention of HIV spread that occurred between the time of joining Maanisha Programme and participation in the study. This was measured using response-based score to a set of seven (7) questions; including: 1) consistent use of condoms with regular partners, 2) consistent use of condoms with non-regular partners, 3) what FSWs do if a client declines to use a condom, 4) consistent use of condoms with clients after joining Maanisha, 5) reduced sexually transmitted infections after joining Maanisha, 6) reduced number of sex clients since joining Maanisha, and 7) not depending fully on sex work as a source of living since joining Maanisha. Similarly, level of knowledge on HIV and AIDS was measured based on a three-point score (poor, moderate and good) to a set of 10 knowledge questions (four on mode of transmission and six on preventive measures). Correct responses were awarded 1 point while incorrect responses elicited a zero point. Questions on knowledge on the modes of HIV transmission included in the study were; 1) having unprotected sex, 2) having multiple sex partners, 3) sharing of infected blood and blood products, and 4) mother-to-child transmission. Further, the HIV and AIDS knowledge level among FSWs was measured based on methods of prevention including; 1) abstinence, 2) being faithful, 3) use of condoms, 4) PMTCT, 5) avoiding sharing of sharps, and 6) going for HIV testing. Further, numerical socio-demographic variables (age, duration in sex work and involvement in AMREF's project) were measured in complete years while the responses to categorical variables (education, religion and marital status) were assigned to appropriate categories. Finally, engagement in alternative economic activities was measured using binary responses to two questions; 1) depended fully on sex work before participating in Maanisha Programme, and 2) currently engaged in an alternative IGA.

*A cross-sectional survey was conducted to measure behaviour change among FSWs in Western and Nyanza provinces in Kenya between June and July 2010. The recruitment of respondents was done through fifteen groups of FSWs participating in the Maanisha Programme. Respondents were eligible for participation if they: 1) were female sex workers; 2) a member of a group participating in the Maanisha Programme; 3) had participated in the Maanisha Programme for at least six months; and 4) provided informed consent. A structured interview schedule was administered to 159 FSWs by trained interviewers. Contacting of study FSWs was done through their group leaders. The questions covered included: socio-demographic characteristics, knowledge of HIV and AIDS, perceptions on risk to HIV infection, positive behaviour change accruing from participation in the Maanisha Programme*.

Binary logistic regression where backward conditional method was specified in order to identify confounders and effect modifiers was used to perform the analysis. The adjusted odds ratios (adj. OR) with their respective 95% confidence intervals (CI) were used to estimate the strength of association between the retained independent variables (parsimonious model) and behaviour change. The study was approved by the AMREF Ethics and Scientific Review Committee (ESRC). In addition, confidentiality was assured by removing all the identifiers prior to data analysis and report writing.

## Results

### Socio-demographic characteristics

The final analysis focused on 159 female sex workers (FSWs) participating in the AMREF Maanisha Programme sites located in Nyanza and Western provinces of Kenya. The average age of the respondents was 31.4 years with a median and mode of 30 and 24 years, respectively. Their age range was 15 to 55 years. The level of education was moderate, with slightly over a half (53%) of the FSWs having attained secondary level education and above. The majority (72%) were Protestant. Six (6) in every ten had at one time been married, with 3% were still in a marital union. Among the formerly married, 20% had been widowed and 19% were either divorced or separated. Only 6% had no children. The mean number of children was 2.6. The minimum number of children ever had was zero, while the maximum was 10. Four (4) in ten FSWs had been in the sex trade for more than five years compared to 57% who had been in it for less than five years (mean: 5.4 and range: <1-25 years).

**Figure 3:**
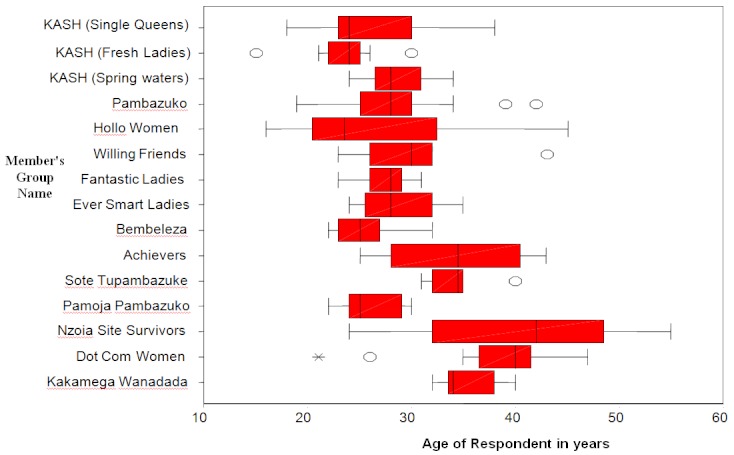
Age of female sex workers by group

The majority (84%) of study FSWs had participated in the Maanisha Programme for at least a year with a mean of 4.4 and range: 1-4 years. Almost three-quarters (73.4%) of the FSWs had not changed their town of residence, while 26.6% had moved mainly from Nairobi, Mombasa, Nakuru, Eldoret and Naivasha towns where they first began engaging in the sex trade. The respondents were involved in supporting the distribution of condoms (79.4%), peer education (75.5%), income generating activities (47.7%) and care and support (22.8%), all components of the Maanisha Programme. The FSWs were drawn from 15 community-based organisations formed based on their age distribution and location. Two groups had a median age of over 40 years with the exception of isolated outliers as illustrated in Figure 3.

### Reasons for engaging in sex work

**Figure 4:**
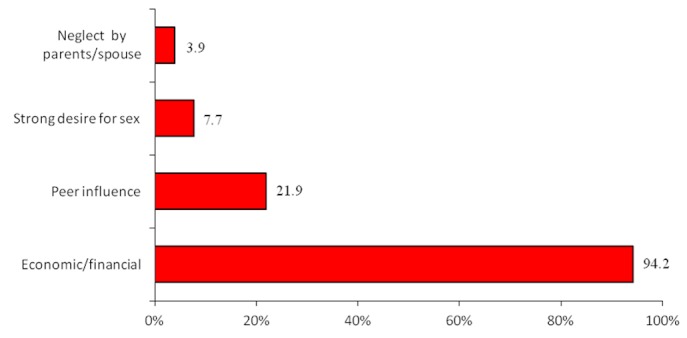
Reasons for engaging in sex work

The respondents were asked to give reasons for choosing commercial sex work. Their responses ranged from social, economic to behavioural and personal reasons. The economic constraint as a “push“ factor into commercial sex work was cited by 94% of the study FSWs. This was distantly followed by peer influence, strong desire/urge for sexual intercourse, and neglect/rejection by parents or husband (Figure 4).

### Behaviour change among FSWs

Behaviour change among FSWs was the central focus of the study. It was analysed through the respondent's adoption level to a set of safer sex practices including; consistent condom use, prompt treatment of STIs and reduction of sex clients. Consistent condom use with non-regular clients was declared by 74.8% of the respondents, 21.1% were inconsistent and 4.1% did not use condoms at all. The proportion of consistent condom use dropped to 64.2% with regular clients while 24.5% were inconsistent and 11.3% never used condoms with regular clients. The respondents were further asked to state what they would do if a client refused to use a condom. Refusal to have sex with the client was declared by 73.5% of the FSWs. However, 15.5% indicated that they charged higher fees, while another 9.7% either did not know what to do or did nothing. Additionally, one FSW reported that she would engage in anal sex while another two indicated they will confess their HIV positive status as a negotiation strategy. Nineteen FSWs (11.9%) revealed that they were HIV positive. Three other FSWs reported that they would insert the female condom if the clients declined to use a male condom. Regarding the average number of sex clients per week, s ranged from 0 to 21, with a median of 5.

**Figure 5:**
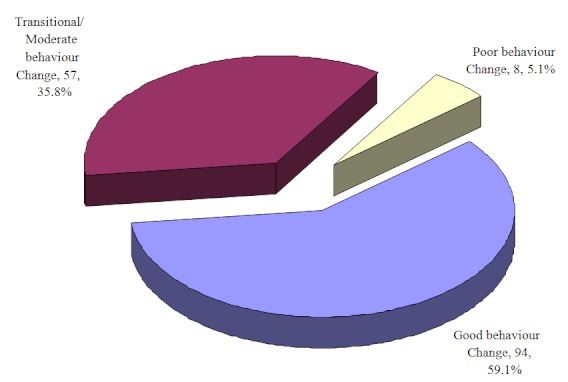
Level of behaviour change among female sex workers

Ultimately, the median score on overall behaviour change was 10 points out of a possible 14. Thirty-two (32) FSWs scored the maximum 14 points compared to one who scored 3 points or below. The results indicate that 59.1% of the FSWs reported good health behaviour change practices by scoring 10-14 points while 35.8% were at the transitional stage, scoring 6-9 points. However, 5.1% of the FSWs exhibited no change in behaviour having scored an aggregate of less than 6 points (Figure 5). In subsequent analysis, the poor and transitional/moderate categories were combined into one segment that accounted for 40.8%, mainly because they were both at risk of HIV infection.

Binary logistic regression was used to model behaviour change (0=poor/moderate, 1=good) using six candidate predictive factors, namely: place of residence (1=Western Province, 2=Nyanza Province), age of the respondent (1= above 30 years, 2= 30 years and below), level of education (1=secondary/ college, 2=none/ primary), religion (1=other, 2=Protestants, 3=Catholic), years with Maanisha (1=2 years and above, 2= below 2 years), period involved in sex work (1= more than 4 years, 2= 4 years and below). These factors were significantly associated (independently) with behaviour change at bivariate analysis. Five successive iterations were performed using backward conditional method in order to eliminate confounders and effect modifiers. Four factors were identified to be the independent predictors of behaviour change. The resulting parsimonious model is shown in Table 1.

**Table 1:** Logistic regression predicting behaviour change using level of education, religious affiliations, period involved in commercial sex work, knowledge of prevention of HIV and AIDS and engagement in alternative income generating activities

Predictor variables	B	s.e. (β)	Adj. OR	95% C.I. for adj. OR		p value
				Lower	Upper	
**Constant**	-2.81	0.67	0.06			<0.001
**Level of education^1^**						
** Secondary/tertiary**	0.80	0.38	2.23	1.06	4.69	0.035*
**Religion affiliation^2^**						0.004*
** Other**	1.53	0.85	4.61	0.87	24.27	0.072
** Protestant**	1.62	0.49	5.07	1.95	13.16	0.001*
**Period involved in CSW^3^**						
** > 4 years**	0.86	0.38	2.36	1.12	5.00	0.025*
**Knowledge on prevention^4^**						0.032*
** Good**	1.47	0.63	4.37	1.26	15.11	0.020*
** Moderate**	0.71	0.39	2.04	0.95	4.38	0.068
**Engagement in IGA^5^**						
** Engaged**	0.83	0.43	2.30	0.98	5.38	0.054

1 Reference category used= “**< Secondary**”

2 Reference category used=“**Catholic**”

3 Reference category used=“**≤** **4 years**”

4 Reference category used=“**Poor**”

5 Reference category used=“**Not engaged**”

* Significant at 0.05 level

Adjusting for religious affiliation, period of involvement in FSW, knowledge on prevention of HIV and AIDS and engagement in alternative IGA, level of education was significantly associated with the FSWs’ behaviour change (p=0.035). A sex worker with secondary/tertiary level of education was 2.32 times more likely to attain good behaviour change compared to one with no/ primary level of education.

Adjusting for level of education, period of involvement in sex work, knowledge on prevention of HIV and AIDS and engagement in alternative IGAs, there was a significant association between religious affiliation and FSWs’ behaviour change (p=0.004). Considering Catholic as the reference category, FSWs’ affiliated to protestant faith were 5.07 times more likely to attain a positive behaviour change compared to those affiliated to the Catholic faith (p=0.001).

Adjusting for level of education, religious affiliation, knowledge on prevention of HIV and AIDS and engagement in alternative IGAs and period of involvement in sex work was significantly associated with FSWs’ behaviour change (p=0.025). FSWs with more than four years in sex work were 2.36 times more likely to attain good behaviour change compared to those with less than four years.

Adjusting for level of education, religious affiliation, period of involvement in sex work and engagement in alternative IGAs, knowledge on prevention of HIV and AIDS was significantly associated with behaviour change (p=0.032). Considering “poor” to be the reference category, clients with moderate knowledge on prevention of HIV and AIDS were 2.04 times more likely to attain a positive behaviour change compared to those with poor knowledge (p=0.068). The likelihood increased to a significant 4.37 for those with good knowledge on prevention of HIV and AIDS (p=0.020).

Adjusting for level of education, religious affiliation, period of involvement in sex work and knowledge on prevention of HIV and AIDS, there was a marginal significant association between engagement in alternative IGA and FSW's behaviour change (p=0.054). FSWs engaged in alternative IGAs were 2.30 times more likely to attain good behaviour change compared to those not engaged in any other alternative IGA.

This multiple logistic regression model presented can be expressed by the following function:

Log “Good behaviour change” = a + β_1_(A: Secondary/Tertiary) + β_2_(B: Other) + β_3_(B: Protestant) + β_4_(C: > 4 years) + β_5_(D: Good) + β_6_(D: Moderate) + β_7_(E: Engaged)

Log “Good behaviour change” = -2.81 + 0.80*(A: Secondary/Tertiary) + 1.53*(B: Other) + 1.62*(B: Protestant) + 0.86*(C: > 4 years) + 1.47*(D: Good) + 0.71*(D: Moderate) + 0.83*(E: Engaged)

Thus;

“Good behaviour change” = (e)^-2.81^ × (e)^0.80*(A: Secondary/Tertiary)^ × (e)^1.53*^(B: Other) × (e)^1.62*(B: Protestant)^ × (e)^0.86*(C: > 4 years)^ × (e)^1.47*(D: Good)^ × (e)^0.71*(D: Moderate)^ × (e)^0.83*(E: Engaged)^

## Discussions

Poverty and personality issues were mentioned as the main reasons that pushed respondents to take up sex trade as a source of livelihood. These reasons for engaging in commercial sex work were similar to those found in previous studies [20, 21–23]. Any efforts to reduce uptake of sex work should aim at addressing such push factors.

This study presents an exploration on behaviour change among female sex workers participating in AMREF's Maanisha Programme in Kenya. Among the respondents interviewed 59% (n=94) were considered to have adopted positive health behaviour practices. This level compares favourably with the 40% level found in the Democratic Republic of Congo [10] and unfavourably with the 95% of CSWs (commercial sex workers) reported in Senegal [9]. Lack of baseline statistics makes it impossible to compare the proportion of FSWs with good behaviour practices at initiation of the Maanisha Programme.

The behaviour change indicator used in this study was constructed based on responses to 14 questions related to changes in behaviour upon initiation and participation in the Maanisha Programme. The definition of behaviour change was restricted to the information collected as responses to the questions asked in the interview schedule. No additional information was collected concerning the sex partners, except on what the FSWs would do when their clients refuse to use a condom. The influence of alcohol and drug use among the FSWs was unexpectedly not captured, yet it is instrumental in determining safer sex practices [24]. It was therefore not possible to determine whether there was an association between alcohol use and adoption of positive health behaviour change. Further, the power of negotiation for safer sex practices with both regular and non-regular partners, especially among the HIV-positive FSWs was not adequately taken into account. This is pivotal in influencing behaviour change in issues of safer sex practices [25].

A number of socio-demographic characteristics were associated with behaviour change in this study. However, unexpectedly, age was not one of them. A study on determinants of consistent condom use in the Democratic Republic of Congo found that consistent use of condoms was associated with age and FSWs aged 20-44 years were more likely to be consistent users [10]. The present study, however, confirms the positive association between duration in commercial sex work and behaviour change that was similarly found in the DRC study. It was striking to note that 3% of the FSWs were still in a marital union. It will be interesting to find out how this group of FSWs managed to continue with the sex trade and maintain their families.

As far as the FSWs’ knowledge on HIV and AIDS was concerned, there exists better knowledge on the modes of transmission of the virus than on prevention. Peer education is a key component of the Maanisha Programme. Other studies found that the peer education strategy played a key role in providing adequate knowledge for behaviour change among FSWs [15, 23]. It was surprising that the FSWs were conversant with the modes of HIV transmission, but not the range of preventive measures. This means there is a gap in the peer education programme of the Maanisha initiative. Condom use is the main preventive measure known by the FSWs. This may be partly because of the encouragement of FSWs to use and distribute condoms over other preventive measures. This finding is comparable to the study in Malawi in which CSWs through their peer education project were encouraged to use and distribute condoms [15]. Further, whereas the peer education activities are in place targeting FSWs, the mass media, especially radio, was cited by majority of respondents as a key source of HIV and AIDS information.

In the context of safer sex practices, the FSWs like in previous studies promote condom distribution and their use, as well as reduced sex partners [9, 10, 15, 26]. Having a quarter of the FSWs using condoms inconsistently with sex partners is a clear avenue for increased spread of HIV. Equally, positive attributes such as the FSWs who would opt to use the female condoms when their male partners were reluctant to use the male condom is something that should be supported and encouraged. It is an approach that can increase consistent use of condoms with all sex partners resulting in the potential reduction of unprotected sex among sex workers [26]. Conversely, 15% of FSWs charged more for those clients who refused to use a condom. While this appeared an option chosen by a significant proportion of study FSWs, it has high health risks and ramifications, especially on increased spread of HIV. Further, anal sex was mentioned as an alternative offer to clients who declined to use condoms. This equally increases the chances of HIV infections as previous studies among men who have sex with men had found [25, 26]. Measures that aim to correct such misinformation among FSWs participating in the Maanisha Programme are recommended.

Substantial amount of resources had been directed towards the provision of capacity to starting alternative income generating activities as sources of income for the FSWs. This was in terms of vocational training (skills development and re-orientation towards an IGA mindset) and the provision of start-up inputs including equipment and money. Use of the same strategy to reach out to the FSWs with alternative means of obtaining income for their livelihood has been implemented successfully elsewhere [20, 27]. However, the effectiveness and sustainability of such interventions need to be understood through further research.

## Conclusion

Substantial health behaviour change has been achieved among some FSWs (59.1%) participating in the Maanisha Programme. The logistic regression model attests that behaviour change was associated with level of education, religious affiliation, number of years in sex work, knowledge on prevention of HIV, and engagement in alternative IGAs. The study thus rejects the null hypothesis that FSWs’ socio-demographic characteristics, knowledge on HIV and engagement in alternative IGAs has no association with behaviour change. The peer education initiative needs to re-orient its focus to include preventive measures, and go beyond the importance of condoms. Currently, FSWs are provided with male condoms whose use largely depends on the male partner's co-operation. It is therefore, recommended that the FSWs be provided with female condoms to ensure consistent use of condoms for safer sex practices and behaviour change. Every effort needs to be made to prevent those already infected (11%) from re-infection. There is need to understand how the children of FSWs cope with their mothers’ lifestyles. The fact that some of the FSWs were currently married calls for a re-orientation of the Maanisha Programme design in addressing behaviour change among this subset of FSWs. This may entail drifting away from peer-to-peer education to couple counselling on the dangers of commercial sex.
